# Neuroanatomical dysmorphology of the medial superior olivary nucleus in sudden fetal and infant death

**DOI:** 10.3389/fnhum.2012.00322

**Published:** 2012-11-27

**Authors:** Anna M. Lavezzi, Luigi Matturri

**Affiliations:** Department of Biomedical, Surgical and Dental Sciences, “Lino Rossi” Research Center for the Study and Prevention of Unexpected Perinatal Death and SIDS, University of MilanMilan, Italy

**Keywords:** sudden infant death syndrome, sudden intrauterine unexplained death syndrome, medial superior olivary nucleus, superior olivary complex, neuropathology

## Abstract

This study expands our understanding of the organization of the human caudal pons, providing a morphologic characterization of the medial superior olivary nucleus (MSO), component of the superior olivary complex (SOC) that plays an important role in the processing of acoustic information. We examined victims of sudden unexplained fetal and infant death and controls (*n* = 75), from 25 gestational weeks to 8 months of postnatal age, by complete autopsy and in-depth autonomic nervous system histological examination, particularly of the MSO nucleus, the focus of this study. Peculiar cytoarchitectural features of the MSO nucleus were found in sudden death cases, such as hypoplasia/agenesis and immature hypercellularity, frequently related to dysgenesis of contiguous structures involved in respiratory rhythm-generating circuit, in particular to hypoplasia of the retrotrapezoid and the facial nuclei. We propose the involvement of this nucleus in more important functions than those related to hearing, as breathing and, more extensively, all the vital activities. Besides, we highlight the fundamental role of the maternal smoking in pregnancy as etiological factor in the dysmorphic neuroanatomical development of the MSO nucleus.

## Introduction

In the course of our neuropathologic investigations on victims of sudden intrauterine unexplained death syndrome (SIUDS) and sudden infant death syndrome (SIDS), we have highlighted specific abnormalities of nuclei and/or structures mediating vital functions, mainly located in the brainstem (Matturri et al., 2002; Lavezzi et al., 2004, 2010, 2012a,b; Matturri and Lavezzi, 2007; Lavezzi and Matturri, 2008a). These neurodevelopmental defects could play a key role in the pathogenetic mechanism of sudden and unexplained perinatal death.

Recently, our attention has been focused on the cytoarchitecture of important neuronal formations assembled in a limited area of the caudal pons that are involved in the respiratory rhythm-generating circuit. In particular we have shown in these pathologies alterations of the facial/parafacial complex (F/PFc) and of the adjacent retrotrapezoid nucleus (RTN) (Lavezzi and Matturri, 2008b; Lavezzi et al., 2012a,b).

The aim of the present study was to expand our understanding on the organization of this brainstem compartment by evaluating whether a further bordering structure, the superior olivary complex (SOC), may somehow be involved in the same developmental pattern and undergo disarranging in SIUDS and SIDS.

The SOC is known as a prominent component of the auditory system being committed to integrate inputs arising from the cochlear nuclei (Moore, 1987; Heffner and Masterton, 1990; Schofield, 2002). Experimental studies (Ollo and Schwartz, 1979; Schofield and Cant, 1991) and researches in adult humans (Kulesza, 2007, 2008) have identified with the same terminology some nuclei into this complex: the “lateral superior olivary nucleus,” the “medial superior olivary nucleus” and a large number of smaller cell groups defined as “periolivary nuclei.”

Here, we provide a morphologic characterization of the human SOC, in particular of its predominant component, the medial superior olivary nucleus (MSO), in perinatal life (75 subjects aged from 25 gestational weeks to 8 postnatal months, died of both known and unknown causes) and discuss pathological details of this nucleus found in victims of SIDS (30 cases) and SIUDS (24 cases).

## Materials and methods

### Study subjects

The cases included in this work are distributed into three groups: 24 cases of SIUDS (mean age 38 gestational weeks/gws, range 25–41 gws), 30 cases of SIDS (mean age 3.7 months, from 1 to 8 months), and 21 control cases (9 fetuses: mean age 36 gws, from 25 to 40 gws; 12 infants: mean age 3.4 months, from 1 to 8 months) who died suddenly but in whom a complete autopsy established an anatomic cause of death. The methods for the histological procedure of the autonomic nervous system, particularly of the brainstem and cerebellum, were those previously reported by us (Matturri et al., 2005, 2008).

Briefly, after fixation in 10% phosphate-buffered formalin, the brainstem and cerebellum were processed and embedded in paraffin. Transverse serial sections were removed from the midbrain, pons, medulla oblongata and cerebellum at intervals of 0.5–1.5 cm (in relation to the victim age). From each sample, 15 levels were obtained and from each level, twelve 5 μm sections, three of which were stained for histological examination using hematoxylin-eosin, Klüver-Barrera stains and Bielchowsky's silver impregnation technique, respectively. The remaining sections were stained/immunostained as deemed necessary or saved for further investigations.

### Histological examination

Routine microscopic evaluation of the brainstems was focused on the hypoglossus, the dorsal motor vagal, the tractus solitarius, the ambiguus, the inferior olivary complex, the caudal raphé, the arcuate nuclei and the pre-Bötzinger complex in the medulla oblongata; on the locus coeruleus, parabrachial/Kölliker-Fuse complex, rostral raphé nuclei in the cranial pons/mesencephalon; on the F/PFc and RTN in the caudal pons. In addition, particular care was paid to examine the SOC and its components, the focus of this study, following the published studies on SOC (Ollo and Schwartz, 1979; Schofield and Cant, 1991; Kulesza, 2007, 2008).

Histological examination of the cerebellum included evaluation of cortex layers (external granular layer; molecular layer, Purkinje cell layer and internal granular layer) and of the medullary deep nuclei (dentate nucleus, fastigial nucleus, globose nucleus, and emboliform nucleus).

The observations were carried out by two independent and blinded pathologists. Comparison among the results was performed employing Kappa statistics (Kappa Index—KI) to evaluate inter-observer reproducibility (see Table 1). The Landis and Koch (1977) system of K I interpretation was used, where 0–0.2 is slight agreement, 0.21–0.40 indicates fair agreement, 0.41–0.60 is moderate agreement, 0.61–0.80 is strong or substantial agreement, and 0.81–1.00 indicates very strong or almost perfect agreement (a value of 1.0 being perfect agreement).

**Table 1:** **Results of the brain microscopic examinations carried out by two pathologists (1 and 2) in 54 victims of sudden unexplained death and interobserver agreement percentage (mean value: 85%)**.

**Brain alterations**	**Pathologist 1**		**Pathologist 2**		**Path.1/Path.2 agreement**		**Interobserver agreement (%)**
	**Yes**	**No**	**Yes**	**No (%)**	**Yes**	**No**	
Hypopl./Agenesis MSO	23	31	24	87	20	27	87
Hypopl./Agenesis arcuate nucleus	11	43	11	100	11	43	100
Hypopl./Agenesis pre-Bötzinger nucleus	7	47	4	90	3	46	90
Hypopl./Agenesis raphé nuclei	8	46	12	74	3	37	74
Hypopl./Agenesis inferior olivary nucleus	10	44	4	85	3	43	85
Hypopl./Agenesis parafacial nucleus	9	45	8	79	3	40	79
Decreased number Purkinje cells	15	39	16	80	10	33	80

This analysis revealed a very satisfactory agreement index (0.85). In addition, in case of discordance among the investigators, the slides were reviewed and discussed until the same result was obtained.

### Ethics considerations for the investigation

Ethical approval for this study was granted by the Italian Health Ministry in accordance with Italian Law n. 31/2006 “*Regulations for Diagnostic Post Mortem Investigation in Victims of Sudden Infant Death Syndrome and Sudden Intrauterine Unexpected Death.*” Parents of all subjects (Controls, SIUDS and SIDS) provided written informed consent to both autopsy and anatomopathologic study, under protocols approved by the Milan University, L. Rossi Research Center institutional Review Board.

### Information collection

For every case, a complete clinical history was collected. Additionally, mothers were asked to complete a questionnaire on their smoking habit, detailing the number of cigarettes smoked before, during and after pregnancy. Twenty-two of the 54 SIUDS/SIDS mothers (40%) were active smokers before and during the pregnancy, smoking more than 3 cigarettes/day. The remaining 32 mothers (60%) admitted no history of cigarette smoking. Six of the 21 mothers of the control group (28%) reported a smoking habit. The remaining 15 mothers (72%) were non-smokers.

### Statistical analysis

The statistical significance of direct comparison between the groups of victims was determined using analysis of variance (ANOVA). Statistical calculations were carried out on a personal computer with SPSS statistical software (version 11.0; SPSS Inc., Chicago, IL, USA). The selected threshold level for statistical significance was *p* < 0.05.

## Results

### Morphological examination of the superior olivary complex (SOC)

The SOC is well recognizable in the most caudal transverse histological sections of the pons as a dense roundish area anterior and slightly medial to the F/PFc and the adjacent RTN. Overall, the SOC can be rostrally observed in our cases, depending on age, for nearly 1–2 mm toward the mid-pons.

**Figure 1:**
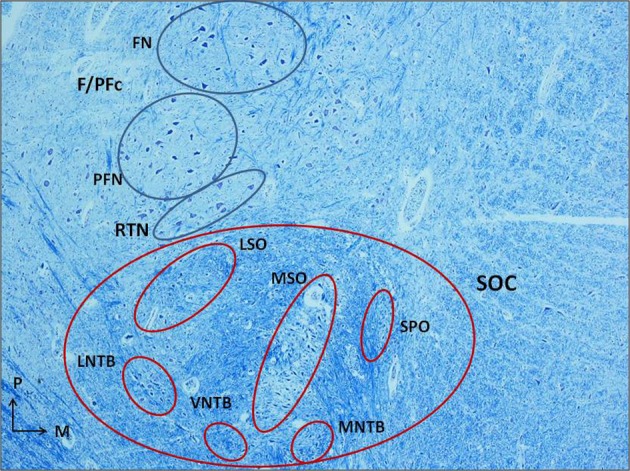
**Histological section of human caudal pons (3 month-old control) showing the localization of the superior olivary complex (SOC), anterior and slightly medial to the facial/parafacial complex (F/PFc), and the adjacent retrotrapezoid nucleus (RTN), and its cytoarchitectural organization.** Klüver–Barrera stain. Magnification: 4×. FN, facial nucleus; F/PFc, facial/parafacial complex; LNTB, lateral nucleus of the trapezoid body; LSO, lateral superior olivary nucleus; MNTB, medial nucleus of the trapezoid body; MSO, medial superior olivary nucleus; PFN, parafacial nucleus; RTN, retrotrapezoid nucleus; SOC, superior olivary complex; SPO, superior paraolivary nucleus; VNTB, ventral nucleus of the trapezoid body.

Even if in perinatal life the process of myelinization is still incomplete, the relative density of myelinized fibers in the SOC region made difficult to us the identification and the examination of its components as defined in literature, although in some cases we have dared to define their boundaries (Figure 1). The only clearly delineated nucleus, given the distinctive rarefraction and peculiar thinness of the fibers in its neuropil, is the MSO nucleus. So, for a comparative and homogeneous analysis, we focused our study on the MSO.

### The medial superior olivary nucleus (MSO) in control cases

The MSO is clearly visible from the 25th gestational week as a compact layer of neurons included in a narrow column with ventral/dorsal orientation.

**Figure 2:**
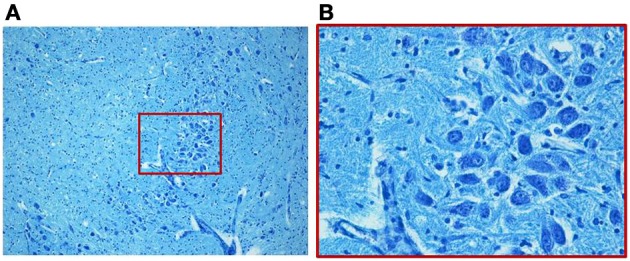
**Photomicrograph of the medial superior olivary nucleus (MSO) in a fetus of the control group died at 28 gestational weeks.** Panel **(B)**, the boxed area of **(A)** at higher magnification, shows a thick group of round immature neurons. Klüver–Barrera stain. Magnification: **(A)** 10×; **(B)** 40×.

**Figure 3:**
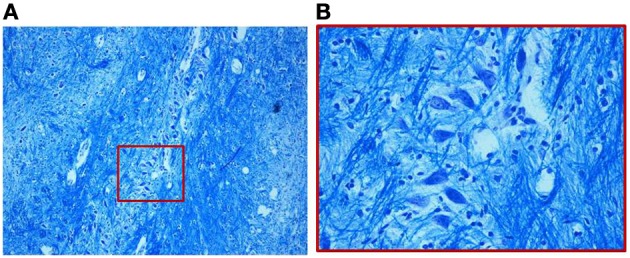
**(A)** Organization of the MSO in a 2-month-old infant of the control group. The neurons, as shown in the magnified boxed area **(B)**, are fusiform with prevalently similar orientation. Klüver–Barrera stain. Magnification: **(A)** 10×; **(B)** 40×.

**Figure 4:**
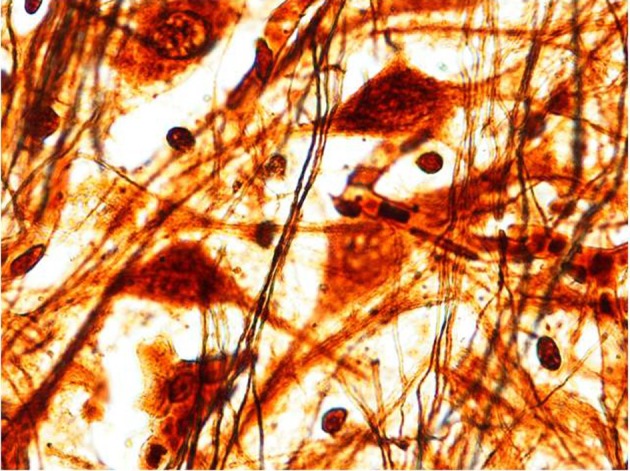
**Multipolar/stellate neurons with broad dendritic arbors in the MSO (2 month-old control).** Bielchowsky's silver impregnation stain. Magnification: 100×.

We find similar cytoarchitecture of the MSO in nearly all subjects of the control group with only some differences in neuronal morphology, orientation and density between fetal and infant deaths. In fetuses around 25–30 gestational weeks we observed a layer of assembled round neurons with rare sketched processes (Figure 2); at the end of pregnancy (from the 35th gestational week) and in postnatal life the neurons were reduced in number with the same orientation, prevalent bipolar fusiform/multipolar stellate in shape, clearly showing dendritic maturation (Figure 3). A broad dendritic arbors of these cell types were well evident in Bielchowsky's stained sections (Figure 4).

Only in 4 control cases (2 fetuses and 2 infants) we highlighted decreased number of neurons with rarefaction of fibers into the MSO.

### The medial superior olivary nucleus (MSO) in siuds and sids cases

**Figure 5:**
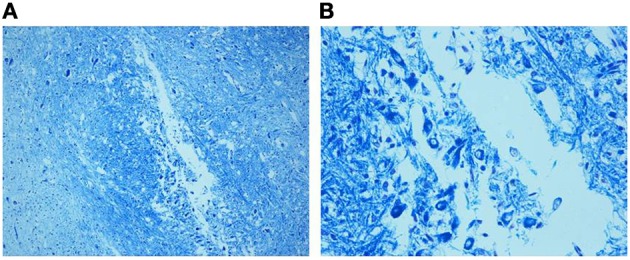
**(A)** Hypoplasia of the MSO (36 gestational week SIUDS case) with lack of fibers and few lateral immature neurons, well visible in **(B)**. Klüver–Barrera stain. Magnification: **(A)** 10×; **(B)** 40×.

**Figure 6:**
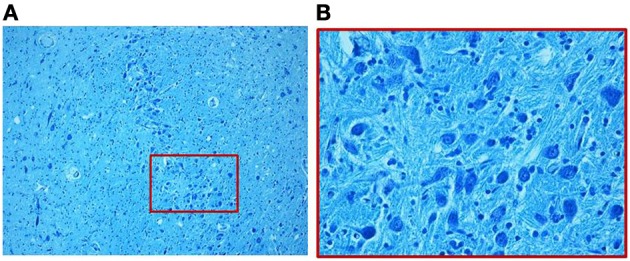
**Dense population of prevalently immature neurons with limited dendritic arbor, as better shown in the boxed area of (A) at greater magnification in (B), in a SIDS case 3-month-old.** Klüver–Barrera stain. Magnification: **(A)** 10×; **(B)** 40×.

Marked disarrangement of the MSO was frequently observed in subjects died of sudden death. In particular, the MSO column was largely devoid of fibers with few scattered immature neurons provided of rare dendrites, frequently distributed on the lateral side of the nucleus, in 7 SIUDS and 9 SIDS cases (MSO hypoplasia) (Figure 5). In addition, in 4 SIUDS it was not possible to recognize the MSO column as expected in the suitable histological sections (MSO agenesis). On the contrary in further 3 suddenly dead infants a very dense population of prevalently immature neurons with limited dendritic arbor and disrupted orientation were found, similar to the ones we typically have seen during fetal stages in control group (Figure 6).

Overall, SIDS and SIUDS cases show a significantly higher incidence of histological alterations of the MSO, as compared with age-matched controls. In fact, 23 of the 54 victims of sudden death (43%), precisely 11 SIUDS and 12 SIDS, but only 4 out of the 21 subjects belonging to control group (19%) showed MSO modifications (*p* < 0.01).

In addition, we observed a significant relation between maternal smoking and developmental disarranging of the MSO. Precisely, 13 of the 22 victims of sudden death with a smoker mother (59%) showed developmental alterations of this nucleus. In the control group, 3 out of the 4 cases with MSO changes had a smoking mother, confirming the detrimental role of prenatal smoke absorption in the pathogenetic mechanism of neuronal defects.

Table 2 summarizes the case profiles of this study, with their relative death diagnosis, the mother's smoking status and the distribution of the MSO alterations.

**Table 2:** **Cases profiles, mother's smoking habit, and distribution of the MSO alterations**.

**Study groups**	**No. of cases**	**Mean age**	**No. of smoking mothers**	**No. of cases with MSO alterations**		
				**Hypoplasia**	**Agenesis**	**Immaturity**
SIUDS	24	38 gws	10	7*^a^*	4*^b^*	–
SIDS	30	3.7 pms	12	9*^c^*	–	3*^d^*
*Fetuses*	9	36 gws	3	2		
CONTROLS	21		6	4*^e^*	–	–
*Infants*	12	3.4 pms	3	2		

gws, gestational weeks; MSO, medial superior olivary nucleus; pms, postnatal months; SIUDS, sudden intrauterine unexplained death syndrome; SIDS, sudden infant death syndrome.

a 6/7 cases had smoking mother.

b 4/4 cases had smoking mother.

c 9/9 cases had smoking mother.

d 3/3 cases had smoking mother.

e 3/4 cases had smoking mother.

### Routine histological evaluation of the brainstem and cerebellum

**Figure 7:**
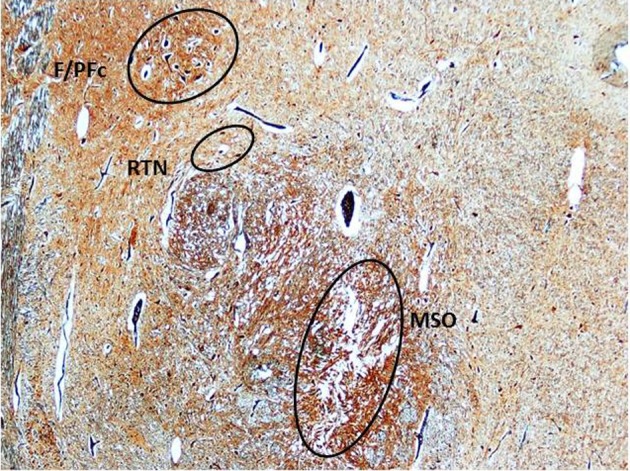
**Triple hypoplasia of MSO, retrotrapezoid nucleus, and facial/parafacial complex in a SIDS victim 2 month-old.** Klüver–Barrera stain. Magnification: 4× F/PFc, facial/parafacial complex; MSO, medial superior olivary nucleus; RTN, retrotrapezoid nucleus.

In both sudden fetal and infant death victims, in addition to the MSO abnormalities, morphological alterations of different brainstem and cerebellar structures (namely hypoplasia/agenesis of the arcuate nucleus, pre-Bötzinger nucleus, inferior olivary nucleus, serotonergic raphé nuclei, parafacial nucleus in the medulla oblongata/pons; decreased number of Purkinje cells in the cerebellum) were disclosed. In particular, Purkinje cell loss was a frequent finding (observed in 9 SIUDS and 6 SIDS). The most frequent association was between MSO agenesis/hypoplasia, hypodevelopment of the adjacent structures (F/PFc and RTN) (Figure 7) and decreased number of Purkinje cells, pointed out in 8 SIUDS victims.

In the control group, hypoplasia of the arcuate nucleus was found in 10 cases.

## Discussion

Our results constitute the first report on the morphological characterization of the human MSO nucleus, component of the SOC known as an important site of convergence of auditorium information from both ears (Moore, 1987; Heffner and Masterton, 1990; Schofield, 2002), in perinatal life and on the pathological aspects of this peculiar structure in SIUDS/SIDS.

Studies on the pathophysiology of SIDS have focused on the brainstem of infants with important results pointing to a reflexogenic lethal mechanism (Rossi and Matturri, 1995). Reflexogenic disorders can in fact lead to inhibitory action on the sinoatrial node with possible cardiac arrest. In particular, a subset of SIDS from sudden loud noises (cardioauditory death) has been reported by Guntheroth (1995). We can suppose in these cases the presence of alterations of brainstem nuclei involved in auditory functions, above all of the MSO, given the high sensitivity of its neurons even to very low-frequency sounds (Yin and Chan, 1990).

The most prominent observation emerged from this study was the loss of neurons and fibers in the MSO region in high percentages of suddenly dead victims.

Nevertheless alterations of the MSO are been reported also in autism, a neurological developmental disorder characterized by cognitive and communicative impairments and restricted repetitive behaviors (Lord et al., 2000; Rice, 2009), in addition to some degree of hearing deficits (Rosenhall et al., 1999; Kellerman et al., 2005).

Kulesza and Mangunay (2008) reported significant differences in the cytoarchitecture of the MSO between autistic individuals and controls, mainly represented by smaller round neurons with shorter dendrites, similar to the MSO neurons found by us in SIUDS and SIDS cases. They interpret the morphological alterations in autistic MSO as related to disfunctions of the auditory pathway. However, we suppose an involvement of the MSO in broader functions than those related to hearing.

Interesting, Rodier et al. (1996) observed a near-complete absence of the superior olive and of the facial nucleus in the brainstem of a 21-year-old autistic woman. This neuropathologic pattern recalls the one present in almost half of the SIUDS victims. In fact, in our study, MSO dysgenesis was frequently associated with hypoplasia of the F/PFc, in addition to the hypodevelopment of the contiguous RTN.

So, we believe that these three adjacent pontine structures are involved in the same pattern of neuronal development. This idea is based on the works of Altman and Bayer (1980) and Chisaka et al. (1992), showing that the facial nucleus and the superior olive not only form at the same embryological stage, but also derive from the same rhombomeres, precisely the fourth and fifth rhombomers. Accordingly, Carpenter et al. (1993) showed that a complete failure of formation of these rhombomers cause lack of the facial nucleus and superior olive in transgenic knockout mice.

Evident demonstrations don't exist in literature that the RTN arises from the same rhombomers. Different investigators refer, however, that neurons located around the facial motor nucleus, called “parafacial respiratory group” (pFRG), together with neurons of the confined RTN, serve as a single pre-inspiratory and/or expiratory-modulating complex in newborns (Onimaru and Homma, 2003; Janczewski and Feldman, 2006). Then it is plausible the involvement of the RTN in the ontogenetic process of the parafacial complex. Moreover, experimental studies on Phox2b expression in rats provide genetic evidence for a similar embryological origin of the two nuclei (Dubreuil et al., 2008).

Since both the F/PFc and the RTN have an essential role in the respiratory rhythm-generating circuit and in maintenance of breathing, we think that also the MSO could have a share in the control of the ventilator activity, in addition to hearing. Our hypothesis is supported by genetic studies showing that the key player in the development of this rhombomeric region of the hindbrain is the proneural transcription factor *mouse atonal homolog 1* (*Math1*). Mice lacking *Math1* lose multiple components of the arousal systems and die shortly after birth from an apparent inability to initiate respiration (Ben-Arie et al., 2000; Wang et al., 2005; Rose et al., 2009).

So, also the alterations of the MSO observed by us in this study and those reported in autism may indicate a disruption of the excitatory/inhibitory eupneic inputs reaching this nucleus, with different degree of severity and pathological consequences, even leading to death in a vulnerable period of perinatal development.

The presence of the triad of abnormalities (MSO, F/PFc, and RTN hypoplasia) confined to victims of sudden fetal death leads to wonder whether breathing alterations can cause death during intrauterine life. It is known that the respiratory-related neuronal network is active before birth checking ventilatory-like rhythmic movements in the mammalian fetus (Barcroft and Barron, 1937; Boddy and Dawes, 1975). Therefore, it becomes vital only after birth. Thus, defects of this occasional respiratory activity in prenatal life would be not sufficient to justify the fetal death. One possibility is that these neuronal structures participate not only in breathing but, more extensively, are essential to the control of all the vital functions.

In autism the MSO disorders are frequently associated with additional anomalies of the brainstem and cerebellum. Increased cell packing density, cell body size decrease with limited dendritic arbor, reduced binding of GABA and nicotinic receptors in different brainstem nuclei and regions and decreased number of cells in cerebellar cortex layers have been reported (Bauman and Kemper, 1985, 2005; Gaffney et al., 1987; Palmen et al., 2004; Kulesza and Mangunay, 2008; Schmitz and Rezaie, 2008). In particular, a reduction in the number of Purkinje cells is considered one of the most consistent pathological feature of autism (Gaffney et al., 1987; Arin et al., 1991; Bauman and Kemper, 1996; Whitney et al., 2008).

The high incidence of Purkinje cell loss in cerebellar cortex and in particular the high correlation with the MSO dysmorphology represents an additional point of convergence between sudden perinatal death and autistic disorders. In addition it enhances our assumption of a MSO involvement in breathing, given the fundamental role of the Purkinje cells in eupneic ventilation (Xu et al., 2004).

As we did in our previous studies (Lavezzi et al., 2005, 2007, 2010, 2012a,b), we ascribe once again the neuropathological findings of this study to smoke absorption in fetal life. Prenatal chronic nicotine exposure primarily alters the development of fetal cholinergic and other neurotransmitter systems, so potentially affecting brainstem centers critical to cardiorespiratory and, more generally, all the autonomic functions control (Duncan et al., 2009). The noxious role of multiple neurotoxins in cigarette smoking during and after pregnancy should be taken into account also in the etiology of autism for which currently underlying biological causes remain to be established.

The conclusion of this work is that considerable remodeling takes place in the MSO of sudden death victims, in addition to neurological alteration of brainstem and cerebellum previously highlighted by us (Matturri et al., 2002; Lavezzi et al., 2004, 2010, 2012a,b; Matturri and Lavezzi, 2007; Lavezzi and Matturri, 2008a). This leads to attribute to the MSO more important functions than those well-known related to hearing. However, this hypothesis should be tested in a larger sample of SIUDS/SIDS for a more detailed understanding of the pathogenesis and neurobiology of these syndromes, and ultimately to more effective preventive interventions, given the relationship with prenatal smoke absorption here reported.

## Author contributions

Anna M. Lavezzi planned the study, analyzed the data, and wrote the manuscript with collaborative input and extensive discussion with Luigi Matturri.

### Conflict of interest statement

The authors declare that the research was conducted in the absence of any commercial or financial relationships that could be construed as a potential conflict of interest.
